# Spatial transcriptomic analysis identifies a necroptosis-prone thyroid follicular cell state in Hashimoto’s thyroiditis

**DOI:** 10.3389/fimmu.2026.1902510

**Published:** 2026-08-03

**Authors:** Dongyu Yang, Cihang Lu, Weiping Teng, Lei Zhao, Xichang Wang, Ying Sun, Haonan Zhang, Shuting Fan, Xiaoguang Shi

**Affiliations:** 1 Department of Endocrinology, Shengjing Hospital of China Medical University, Shenyang, Liaoning, China; 2 Department of Endocrinology and Metabolism and the Institute of Endocrinology, NHC Key Laboratory of Diagnosis and Treatment of Thyroid Diseases, The First Hospital of China Medical University, Shenyang, Liaoning, China; 3 Department of Laboratory Medicine, National Clinical Research Center for Laboratory Medicine, The First Hospital of China Medical University, China Medical University, Shenyang, Liaoning, China

**Keywords:** Hashimoto’s thyroiditis, thyroid follicular cells, necroptosis, RIPK3, MLKL, spatial transcriptomics, TNF-α

## Abstract

**Background:**

Hashimoto’s Thyroiditis (HT) is the leading cause of primary hypothyroidism, characterized by progressive thyroid follicular cell (TFC) loss and diffuse lymphocytic infiltration. While apoptosis dominates TFC death in HT, the role of pro-inflammatory necroptosis in TFC destruction, especially its cellular heterogeneity, spatial distribution, and inflammatory microenvironmental regulation, remains incompletely elucidated.

**Methods:**

We integrated spatial transcriptomics and multilevel functional validation to map necroptosis-associated cellular and molecular programs in HT. Thyroid tissues from a local clinical cohort were analyzed using transmission electron microscopy (TEM), immunohistochemistry, and immunoblotting to validate necroptosis-associated signatures. *In vitro*, Nthy-ori 3-1 cells were stimulated with TNF-α, with or without IFN-γ, to characterize necroptosis-associated signaling in thyrocytes.

**Results:**

We identified a distinct TFC subpopulation (Cluster 2) with high enrichment of the TNF signaling pathway and TNFRSF1A expression, which exhibited a necroptosis-susceptible phenotype and served as the core receiver of inflammatory signals from the immune microenvironment. Pseudotime trajectory analysis showed synchronous upregulation of transcripts of core necroptotic molecules (RIPK3, MLKL) at a critical threshold during TFC dedifferentiation. The TEM revealed necroptosis-compatible ultrastructural features in HT thyrocytes. Notably, HT tissues showed an RIPK3-MLKL-predominant necroptosis-associated pattern, with no significant changes in total or phosphorylated RIPK1. Clinically, the expression of necroptosis-related markers (ZBP1, RIPK3, MLKL) in thyroid tissues and circulating pro-inflammatory cytokines (IL-6, IL-1β, IL-1α) in serum were significantly correlated with thyroid autoantibody (TPOAb, TgAb) titers and TFC dedifferentiation. *In vitro*, pharmacological inhibition of RIPK3 or MLKL effectively preserved thyrocyte membrane integrity, reduced cell death, and suppressed the secretion of pro-inflammatory cytokines.

**Conclusions:**

Our study provides a multimodal characterization of necroptosis in HT, showing that the inflammatory immune microenvironment is associated with TFC loss and activation of an RIPK3-MLKL-predominant necroptosis-related program, without detectable upregulation of total or phosphorylated RIPK1. Targeting the RIPK3-MLKL axis may represent an experimental therapeutic hypothesis that requires further validation in primary thyrocytes, organoids, and *in vivo* HT models.

## Introduction

Hashimoto’s Thyroiditis (HT) is the most common autoimmune thyroid disease (1) and a major cause of hypothyroidism worldwide (2). It involves immune-mediated destruction of thyroid follicular cells (TFCs) (3), yet the exact mechanisms of thyrocyte loss remain unclear. Current treatment relies on hormone replacement rather than targeting the underlying immune dysfunction, underscoring the critical need to elucidate the mechanisms underlying TFC loss.

Historically, thyrocyte loss in HT has been interpreted mainly through apoptosis-related mechanisms, including Fas/FasL signaling, cytotoxic lymphocyte-associated pathways, and cytokine-conditioned death-receptor sensitivity (4–6). However, recent advances in cell death biology have identified necroptosis as a distinct and highly inflammatory form of programmed cell death. Unlike immunologically silent apoptosis, necroptosis is mediated by the receptor-interacting protein kinase 1 (RIPK1), RIPK3, and mixed lineage kinase domain-like protein (MLKL) axis. This programmed cell death process culminates in plasma membrane rupture and the subsequent release of damage-associated molecular patterns (DAMPs) (7). This release triggers innate immune responses, potentially creating a vicious cycle of inflammation and tissue damage. While necroptosis has been implicated in multiple inflammatory disorders such as inflammatory bowel disease (8) and psoriasis (9), its specific activation, spatial distribution, and clinical significance in HT remain largely unexplored.

In this study, we hypothesized that necroptosis, a pro-inflammatory mode of programmed cell death, contributes to TFC loss in HT. Using spatial transcriptomics, and functional validation assays, we identified a necroptosis-prone TFC subset and found that pharmacological inhibition of downstream necroptotic execution pathways attenuates cytokine-induced thyrocyte injury *in vitro*, thereby supporting further evaluation of this pathway as a preclinical target.

## Materials and methods

### Ethical approval and informed consent

This study was approved by the Ethics Committee of Shengjing Hospital of China Medical University (Approval No. 2024PS794K) and was conducted in accordance with the Declaration of Helsinki as revised in 2013. Informed consent was obtained from all individual participants prior to their inclusion in the study.

### Data acquisition and quality control

The spatial transcriptomics data from three HT and two healthy control samples (GEO: GSE248205) were processed and analyzed using Seurat (v4.0) (10). Following standard quality control procedures to filter low-quality spatial capture spots, a total of 9,158 high-quality observations were retained for downstream analysis. Detailed sequencing quality metrics and per-sample cell counts are provided in **Supplementary Table 1**, **Supplementary Figures 1A, B, D, E**, **3**.

### Spatial transcriptomics processing, cell identification, and intercellular communication

The data were processed using the Seurat (v4.0) R package. Dimensionality reduction was performed via RunPCA, followed by unsupervised clustering using FindClusters with a resolution of 0.5 based on the top 30 principal components. After visualization with RunUMAP, clusters were annotated using established lineage markers (**Supplementary Tables 2**, **3**, **Supplementary Figure 2**). Subsequently, the CellChat tool was employed to reconstruct the intercellular communication network within the thyroid microenvironment. By integrating expression profiles with a curated ligand-receptor database, we calculated the communication probability (probabilistic strength) between various cell types to identify key signaling axes in HT.

### Spatial module-score analysis in HT sections

Module-score analysis was performed to characterize the correlation between inflammatory activity, thyroid follicular cell differentiation, and necroptosis signaling in HT. The analysis was performed in three HT sections from dataset GSE248205 (HT1, HT2, HT3) and validated in two independent HT sections (P3, P4) from GSE230424. Spatial count matrices were processed as AnnData objects. After quality filtering (retaining tissue-area spots and removing genes detected in <3 spots), raw counts were normalized to 10,000 counts per spot and log-transformed. Highly variable gene selection, dimensionality reduction, and Leiden clustering were performed for quality control and spatial domain assessment.

Four predefined module scores were calculated with consistent gene sets across all sections: thyroid follicular cell differentiation score (TG, TPO, PAX8, SLC5A5, TSHR, NKX2-1, IYD), inflammatory cytokine score (TNF, IFNG, IL1B, IL6, CXCL9, CXCL10, CCL2, CCL5), RIPK3-MLKL execution score (RIPK3, MLKL, ZBP1, JAK2, STAT1, IRF1), and RIPK1-related score (RIPK1, TNFRSF1A, TRADD, FADD, CASP8). Spatial distributions of module scores were visualized by mapping spot-level scores to tissue coordinates or matched H&E images. Spot-level Spearman correlation was applied to assess spatial coupling between biological programs. All analyses were implemented in Python 3 using Scanpy, AnnData, pandas, NumPy, SciPy, scikit-learn, and Matplotlib in a Google Colab environment.

### Transcriptomic integration, functional enrichment, and spatial-temporal trajectory analysis

To identify core molecular contributors to HT progression, differential expression analysis was performed using DESeq2 on two independent public datasets (GSE138198 and HRA001684) (11, 12). Differentially expressed genes (DEGs) were identified based on the criteria of |log2FC| > 0.45 and *P* value < 0.05 (**Supplementary Tables 11**, **12**). The union of these DEGs was then subjected to KEGG pathway enrichment analysis using the clusterProfiler package. Spatially, based on the dataset GSE248205 (**Supplementary Figure 3**) (10), SpatialFeaturePlot was utilized to visualize the localized expression of pro-inflammatory mediators (TNF, IFNG, TRAF5) and the necroptosis executioner (MLKL) within the thyroid tissue. We further calculated the spatial co-expression of these markers with TFCs across capture spots. Finally, Monocle3 was used to reconstruct the pseudotime developmental trajectory. The plot_genes_in_pseudotime function was applied to monitor the expression dynamics of core genes, enabling the identification of critical molecular thresholds driving TFC degeneration.

### Evaluation of biomarker candidates and their associated clinical indicators

The clinical diagnostic utility of necroptosis-related molecules in HT was evaluated using the pROC package. Receiver Operating Characteristic (ROC) curves were constructed, and the Area Under the Curve (AUC) was calculated to quantitatively assess the sensitivity and specificity of candidate genes (e.g., ZBP1, RIPK3, MLKL) in distinguishing HT patients from healthy controls. The AUC value exceeding 0.90 was established as the threshold for a high-accuracy diagnostic biomarker and 0.70 for moderately accurate (13). These analyses were extended to systemic inflammatory markers (e.g., IL-6, IL-1β) to explore their potential for clinical screening and disease monitoring.

### Virtual knockout analysis via scTenifoldKnk

Virtual knockout analysis was conducted using scTenifoldKnk (v1.0.1) (14) to evaluate the regulatory impact of RIPK1 perturbation in thyroid follicular cells (TFCs). A total of 937 TFCs were extracted from the integrated HRA001684 dataset. Gene regulatory networks were reconstructed from the raw count matrix using the top 3,000 highly variable genes supplemented with curated necroptosis-related, inflammatory, and thyroid differentiation-associated genes. RIPK1 virtual knockout was simulated by removing regulatory edges connected to RIPK1, followed by manifold alignment to quantify gene-level perturbation effects. The analysis was repeated across three independent random seeds, and genes with Benjamini–Hochberg-adjusted P < 0.05 in at least two of three runs were defined as robustly perturbed.

### Study population and cell culture models

Thyroid para-carcinoma tissues were collected from euthyroid HT patients with positive serum thyroid autoantibodies and postoperative pathological confirmation of HT, as well as from euthyroid controls with negative thyroid autoantibodies and no pathological evidence of HT. Detailed inclusion/exclusion criteria and cohort characteristics are provided in **Supplementary Tables 13**, **14**. For *in vitro* studies, the human thyroid follicular epithelial cell line Nthy-ori 3-1 was used. Cells were stimulated with TNF-α and IFN-γ to mimic the inflammatory microenvironment, and mechanistic inhibition experiments were performed using inhibitors targeting RIPK3, MLKL, caspases, and IRF1. Detailed culture conditions and reagent information are provided in the Supplementary Materials.

### Morphological, histological, and ultrastructural analysis of thyroid tissues

Hematoxylin and eosin staining was performed to evaluate thyroid morphology, follicular disruption, and lymphocytic infiltration. Immunohistochemistry and immunofluorescence were used to assess the localization of necroptosis-related proteins in thyroid follicular cells. Transmission electron microscopy was performed on thyroid tissues and cell pellets to evaluate ultrastructural features of necroptosis, including organelle swelling and plasma membrane rupture. Detailed staining procedures, microscopy conditions, and antibody information are provided in **Supplementary Table 15** and the **Supplementary Methods**.

### Biochemical and molecular assays

Gene expression was assessed by qRT-PCR, protein expression by immunoblotting, and cytokine levels in serum or cell supernatants by ELISA. Cell viability and cell death were evaluated using CCK-8, LDH release, and Annexin V-FITC/PI flow cytometry assays. Detailed reagent information, assay procedures, and primer sequences are provided in **Supplementary Table 16** and the **Supplementary Methods**.

### Statistics and reproducibility

All data were expressed as the mean ± standard deviation. *In vitro* experiments were independently replicated at least three times. ImageJ software was employed for image-based data analysis. One-way analysis of variance (ANOVA) was used to conduct statistical analysis for multiple group comparisons. For categorical variables, the Chi-square test was used for two-group comparisons. For datasets that failed to conform to the normal distribution, the nonparametric Kruskal-Wallis test was adopted instead. In terms of two-group comparisons, an independent-samples t-test was applied to normally distributed data, while the Mann−Whitney U test was utilized for non-normally distributed data. Statistical analyses and data visualizations were primarily implemented using GraphPad Prism version 9.4.0 (GraphPad Software, San Diego, USA), Python 3, and R software (version 4.2.2; R Foundation for Statistical Computing, Vienna, Austria). Pearson’s or Spearman’s correlation analysis was performed to evaluate the correlation among variables from different experiments. Both * and # were used to indicate statistical significance with identical significance thresholds, while they differed in the comparison objects. Statistical significance was designated as follows: *P < 0.05, **P < 0.01, ***P < 0.001, ****P < 0.0001; #P < 0.05, ##P < 0.01, ###P < 0.001, ####P < 0.0001; ns represented no statistical significance.

### Results

### Remodeling of the cellular landscape in Hashimoto’s thyroiditis

**Figure 1:**
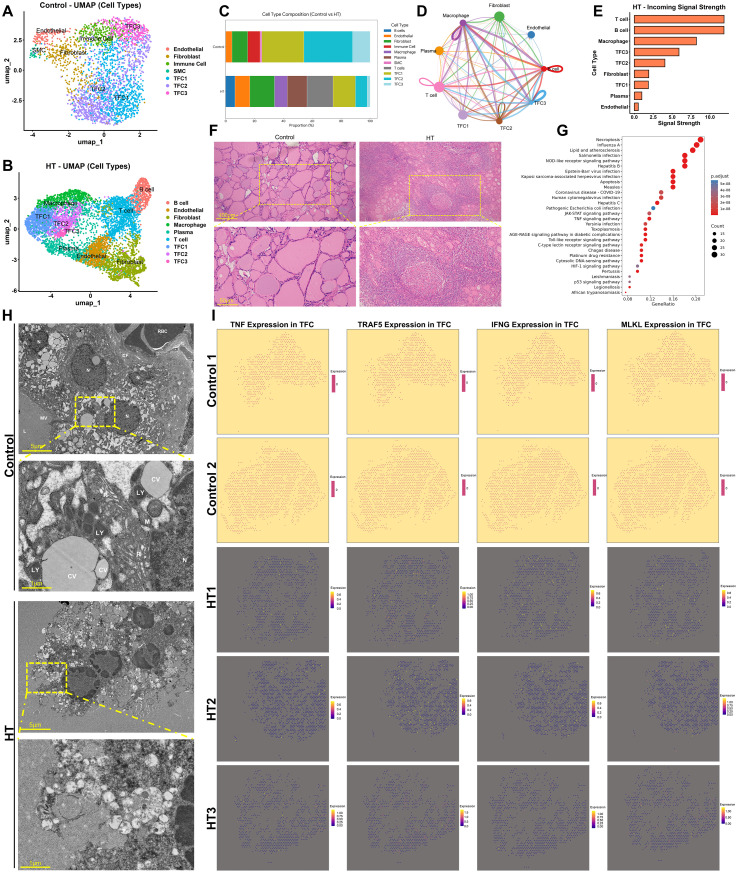
Cellular landscape remodeling, inflammatory signaling, and necroptosis-associated changes in Hashimoto’s Thyroiditis (HT). **(A-B)** UMAP visualization of cellular populations identified from Control **(A)** and HT **(B)** thyroid samples. Cell clusters were annotated according to canonical marker gene expression. **(C)** Relative cellular composition of Control and HT samples. **(D)** Intercellular communication network reconstructed using CellChat, mapping the interactions and signaling fluxes among distinct cell types within the HT microenvironment. **(E)** Bar graph depicting the incoming signaling strength across various cell types in HT tissues. **(F)** Representative Hematoxylin and Eosin (H&E) staining of Control and HT thyroid tissue sections. **(G)** Kyoto Encyclopedia of Genes and Genomes (KEGG) pathway enrichment analysis based on the union of differentially expressed genes (DEGs) from independent transcriptomic cohorts. **(H)** Transmission electron microscopy (TEM) ultrastructural analysis of thyroid tissues. Thyroid follicular cells in the normal group exhibited normal morphology, with apical microvilli (MV) projecting into the follicular lumen (L). The nucleus (N) contained central euchromatin, with peripheral heterochromatin clumps visible at the margin, accompanied by parallel cisternae of rough endoplasmic reticulum (R) and mitochondria (M). Additionally, numerous dense lysosomal (LY) granules and colloidal vesicles (CV) were present. Under low magnification, red blood cells (RBCs), collagen fibers (CF), and wide intercellular spaces were observed, indicating active cellular function. In the HT group, microvilli of thyroid follicular epithelial cells were lost; organelles were swollen and ruptured, the plasma membrane was disrupted, and nuclear chromatin showed condensation and margination. **(I)** Spatial transcriptomics mapping of targeted genes (TNF, IFNG, TRAF5, MLKL) across tissue capture spots.

Analysis of thyroid cellular composition revealed marked remodeling of the thyroid microenvironment in HT. TFCs accounted for 75.0% of cells in controls and 25.7% of cells in HT samples (**Figures 1A–C**; **Supplementary Figures 1C, F**, **Supplementary Tables 2**-**4**). Residual TFCs in HT showed evidence of pathological reprogramming. HT-TFC2 exhibited increased expression of KRT19, whereas HT-TFC3 showed elevated DUOXA2 expression, consistent with an injury-associated and oxidative stress-related state (**Figure 1B**; **Supplementary Table 3**). Histological examination further showed lymphocytic infiltration and fibrosis in HT tissues (**Figure 1F**). CellChat analysis identified TFC3 as a major receiver of inflammatory signals from macrophages, T cells, B cells, and autocrine TFC loops (**Figures 1D, E**; **Supplementary Tables 5**, **6**). Consistently, KEGG enrichment analysis based on the union of DEGs from GSE138198 and HRA001684 showed significant enrichment of necroptosis, apoptosis, and TNF signaling pathways (**Figure 1G**).

### Spatial and ultrastructural confirmation of necroptosis in HT tissues

Transmission electron microscopy (TEM) revealed characteristic necroptotic ultrastructural changes in HT thyrocytes, including plasma membrane rupture, organelle swelling, vacuolization, and chromatin condensation. In contrast, control thyrocytes showed preserved microvilli, organized rough endoplasmic reticulum, and dense lysosomal granules, consistent with intact secretory function (**Figure 1H**).

Spatial transcriptomic analysis further showed colocalization of pro-inflammatory mediators, including TNF, IFNG, and TRAF5, with MLKL in TFC-enriched regions of HT tissues, whereas this pattern was not observed in controls (**Figure 1I**). These findings support localized activation of necroptotic machinery within inflamed thyroid follicular regions.

### Activation of the RIPK3-MLKL Axis in HT patients

**Figure 2:**
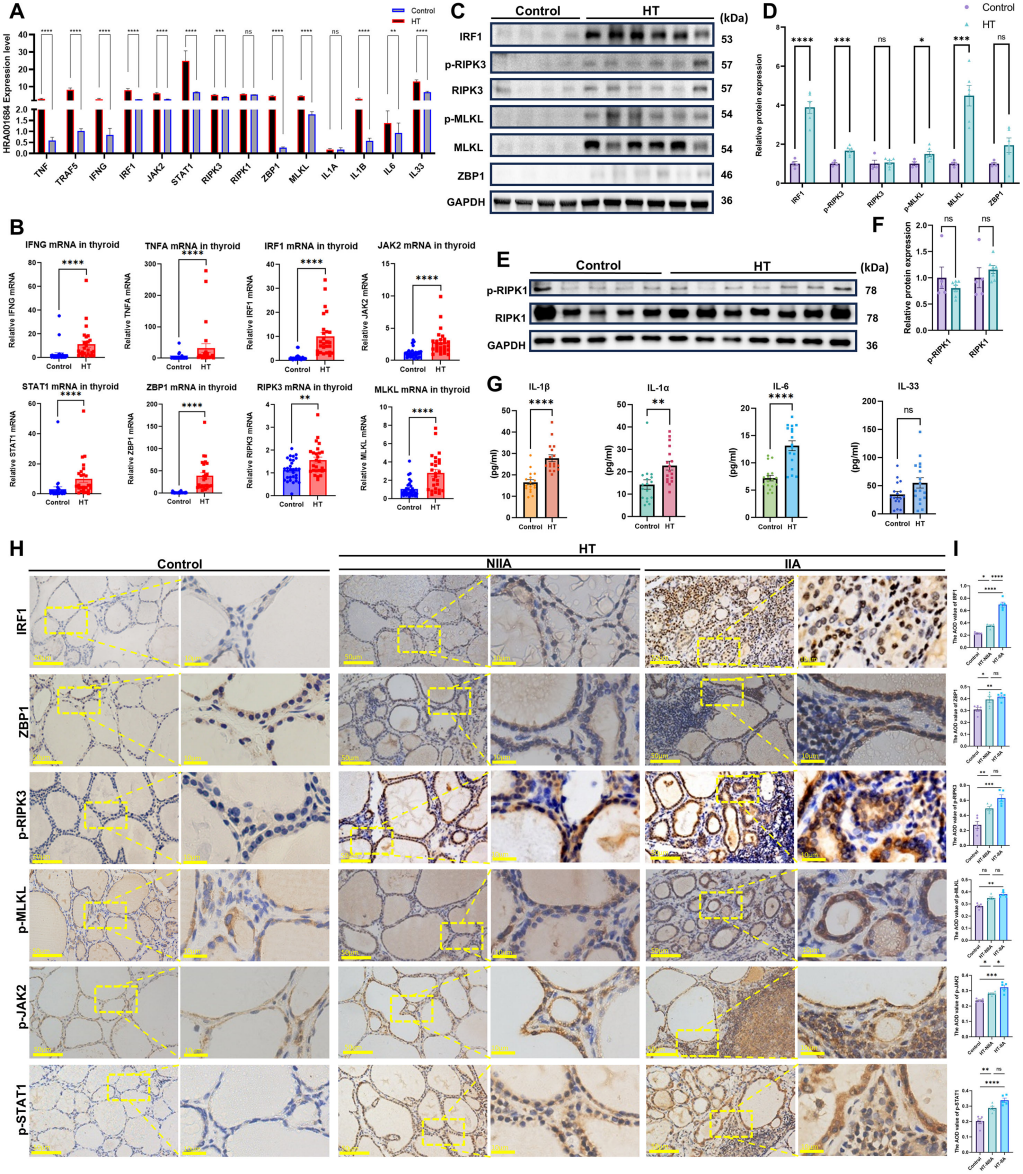
Expression levels and spatial distribution of necroptosis-related markers in Control and Hashimoto’s Thyroiditis (HT) tissues. **(A)** Bar graph showing the relative transcriptomic expression levels of necroptosis-related genes from independent dataset HRA001684 in Control and HT groups. **(B)** Quantitative real-time PCR (qRT-PCR) analysis of IFNG, TNFA, JAK2, STAT1, IRF1, ZBP1, RIPK3, and MLKL mRNA levels in local cohort thyroid tissues. **(C)** Representative immunoblotting images of IRF1, p-RIPK3, RIPK3, p-MLKL, MLKL, and ZBP1 protein levels in Control and HT tissue lysates. GAPDH was utilized as the internal loading control. **(D)** Quantitative densitometry analysis of the relative protein expression corresponding to the western blot results shown in **(C)**. **(E)** Representative immunoblotting images of p-RIPK1 and RIPK1 proteins in Control and HT tissue lysates, with GAPDH as the loading control. **(F)** Quantitative densitometry analysis of the relative protein expression corresponding to the western blot results shown in **(E)**. **(G)** Enzyme-linked immunosorbent assay (ELISA) quantification of serum IL-1β, IL-1α, IL-6, and IL-33 concentrations (pg/mL) in Control and HT patients. **(H)** Representative immunohistochemistry (IHC) images illustrating the *in situ* expression and spatial distribution of IRF1, ZBP1, p-RIPK3, p-MLKL, p-JAK2, and p-STAT1 in Control tissues, as well as in non-immune-infiltrated areas (NIIA) and immune-infiltrated areas (IIA) of HT tissues. Scale bars are indicated in the lower left corner of each respective panel. **(I)** Quantitative analysis of the average optical density (AOD) for the IHC staining shown in **(H)**. Statistical significance is indicated as follows: *P < 0.05, **P < 0.01, ***P < 0.001, and ****P < 0.0001; ns, not significant.

Transcriptomic analysis of independent HT datasets showed broad upregulation of necroptosis-related genes, including ZBP1, JAK2, STAT1, RIPK3, and MLKL (**Figure 2A**; **Supplementary Figure 4**). In our local cohort, qRT-PCR confirmed increased mRNA expression of IFNG, TNFA, JAK2, STAT1, IRF1, ZBP1, RIPK3, and MLKL in HT thyroid tissues (**Figure 2B**).

At the protein level, immunoblotting showed increased IRF1, phosphorylated RIPK3 (p-RIPK3), and phosphorylated MLKL (p-MLKL) in HT tissues (**Figures 2C, D**). In contrast, neither total RIPK1 nor phosphorylated RIPK1 differed significantly between HT and control tissues (**Figures 2E, F**). Although ZBP1 mRNA was elevated, ZBP1 protein in bulk lysates did not reach statistical significance (**Figures 2C, D**).

Immunohistochemistry localized p-JAK2, p-STAT1, IRF1, ZBP1, p-RIPK3, and p-MLKL to thyroid follicular cells, with the strongest staining detected in immune-infiltrated areas relative to non-infiltrated areas and controls (**Figures 2H, I**). In parallel, serum IL-1β, IL-1α, and IL-6 levels were increased in HT patients (**Figure 2G**).

### Spatial module-score analysis supports a RIPK3-MLKL predominant inflammatory pattern

**Figure 3:**
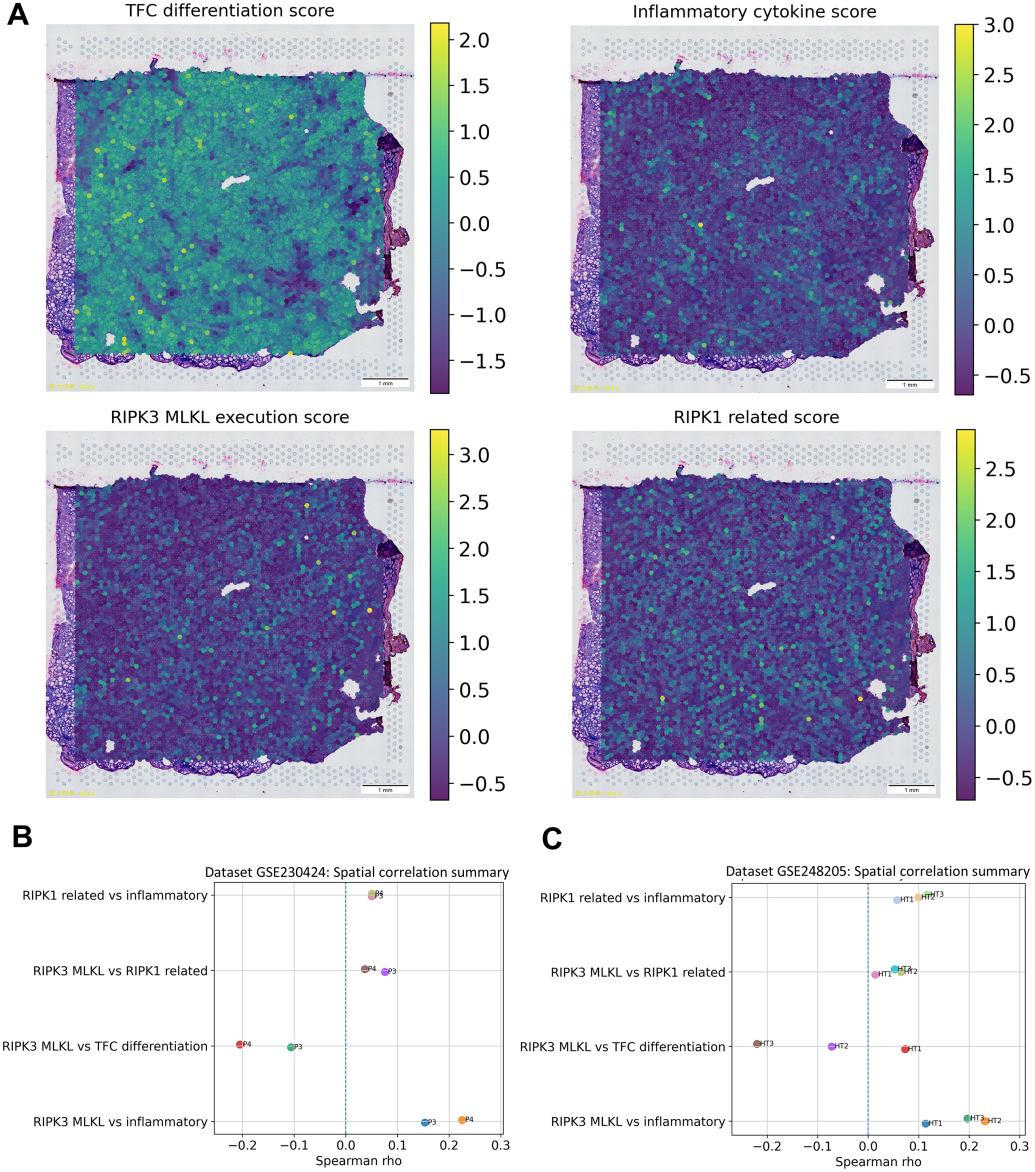
Spatial module-score analysis supports modest inflammatory coupling of the RIPK3-MLKL execution program in Hashimoto’s thyroiditis (HT). **(A)** Representative spatial maps from the P4 HT section in GSE230424 showing the distribution of the TFC differentiation score, inflammatory cytokine score, RIPK3-MLKL execution score, and RIPK1-related score overlaid on the hematoxylin and eosin image. **(B)** Summary of spot-level Spearman correlation coefficients in the two independent HT sections from GSE230424. Each dot represents one HT section. **(C)** Summary of spot-level Spearman correlation coefficients in the three HT sections from GSE248205. Each dot represents one HT section. The vertical dashed line indicates rho = 0.

To further examine whether the RIPK3-MLKL activation observed at the protein level was spatially coupled to inflammatory or RIPK1-related transcriptional programs, we performed module-score analysis in HT sections from GSE248205 and GSE230424 (**Figure 3**, **Supplementary Figures 5**, **6**, **Supplementary Table 7**). Across the five HT spatial sections, the RIPK3-MLKL execution score showed a reproducible but modest positive association with the inflammatory cytokine score. In contrast, its correlation with the RIPK1-related score was consistently weak and close to zero. These findings suggest that the spatial enrichment of the RIPK3-MLKL module is more closely linked to local inflammatory activity than to RIPK1/TNFR1-adaptor transcriptional variation.

### Necroptosis contributes to dedifferentiation and correlates with autoimmunity

ROC analysis showed that necroptosis-related genes, including ZBP1, RIPK3, and MLKL, as well as upstream cytokines such as IFNG and TNFA, had moderate diagnostic value for HT across datasets (AUC > 0.80), whereas serum IL-6 and IL-1β showed high predictive performance (AUC > 0.90) (**Supplementary Figure 7**).

Correlation analysis further demonstrated strong positive associations between inflammatory cytokines and necroptosis-related genes (**Supplementary Figure 8A**). Serum IL-1α, IL-1β, and IL-6 were also positively correlated with one another (**Supplementary Figure 8B**). In contrast, the necroptosis/inflammation signature was negatively correlated with thyroid differentiation markers, including TPO, TG, SLC26A4, and NKX2-1 (**Supplementary Figure 8C**).

**Figure 4:**
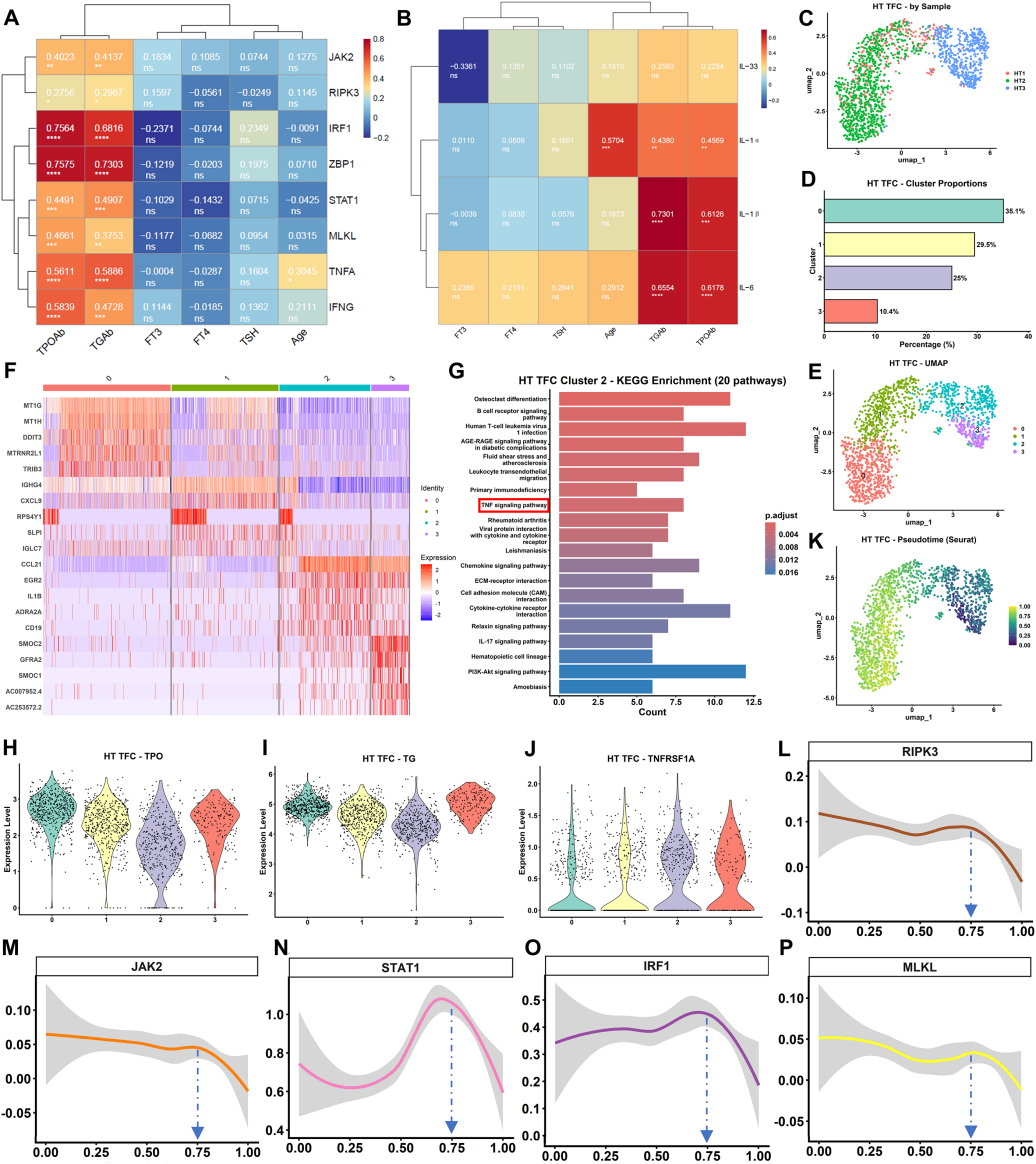
Single-cell pseudotime trajectory and clinical correlation analysis of the thyroid follicular cell (TFC) compartment. **(A-B)** Correlation matrices illustrating the relationship between the expression of necroptosis-related genes **(A)**, serum cytokines **(B)**, and clinical parameters, including thyroid autoantibody titers (TPOAb, TgAb) and thyroid function markers (FT3, FT4, TSH). Color gradients indicate the correlation coefficients. **(C)** Uniform Manifold Approximation and Projection (UMAP) plot displaying the distribution of TFCs derived from HT samples, color-coded by sample origin (HT1, HT2, HT3). **(D)** Bar chart illustrating the proportional distribution of the four identified TFC subclusters (Clusters 0–3) within the HT TFC compartment. **(E)** UMAP plot of the HT TFC compartment, color-coded to identify the four distinct TFC subclusters. **(F)** Heatmap depicting the expression patterns of the top marker genes across TFC Clusters 0–3. **(G)** Bar plot showing the top 20 enriched pathways from the Kyoto Encyclopedia of Genes and Genomes (KEGG) analysis for TFC Cluster 2. **(H-J)** Violin plots displaying the expression distributions of TPO **(H)**, TG **(I)**, and TNFRSF1A **(J)** across the four TFC subclusters. **(K)** Pseudotime trajectory of HT-derived TFCs projected onto the UMAP space, colored by pseudotime progression from dark blue (early) to yellow (late). **(L-P)** Line plots illustrating the dynamic expression trends of RIPK3 **(L)**, JAK2 **(M)**, STAT1 **(N)**, IRF1 **(O)**, and MLKL **(P)** along the pseudotime trajectory. The dashed blue arrows indicate the alignment of peak expression at pseudotime point ~0.75.

At the clinical level, tissue expression of TNFA, IFNG, IRF1, JAK2, STAT1, RIPK3, MLKL, and ZBP1, together with serum IL-1α, IL-1β, and IL-6, was significantly correlated with TPOAb and TgAb titers (**Figures 4A, B**). These findings link necroptosis-related signaling to both thyrocyte dedifferentiation and autoimmune activity in HT.

### Trajectory analysis identifies a necroptosis-prone TFC subpopulation

To define the temporal progression of TFC injury, we performed pseudotime analysis on HT-derived TFCs. Re-clustering identified four TFC subclusters, among which Cluster 2 displayed the lowest expression of differentiation markers such as TPO and TG and the strongest enrichment of the TNF signaling pathway (**Figures 4C–I**). Cluster 2 also showed the highest expression of TNFRSF1A, encoding the major TNF death receptor TNFR1 (**Figure 4J**).

**Figure 5:**
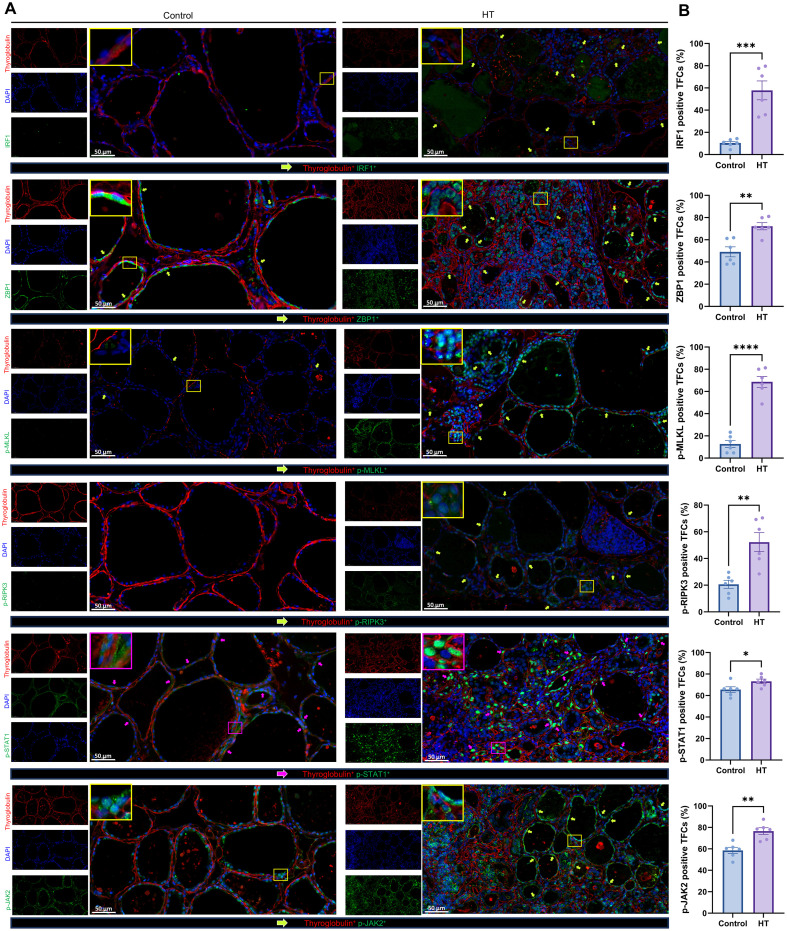
*In situ* immunofluorescence validation of necroptosis-related proteins in thyroid follicular cells (TFCs). **(A)** Representative double immunofluorescence images of Control and HT thyroid tissue sections. Tissues were co-stained for Thyroglobulin (red) to specifically label TFCs, and target proteins (IRF1, ZBP1, p-MLKL, p-RIPK3, p-STAT1, and p-JAK2; green). Nuclei were counterstained with DAPI (blue). The boxed areas indicate magnified regions highlighting the cellular colocalization of the target proteins with Thyroglobulin-positive TFCs. Scale bars: 50 μm. **(B)** Bar graphs presenting the statistical quantification of the immunofluorescence staining, calculated as the percentage of target protein-positive TFCs relative to total Thyroglobulin-positive TFCs in the Control and HT groups. Statistical significance is indicated as follows: *P < 0.05, **P < 0.01, ***P < 0.001, and ****P < 0.0001; ns, not significant.

Pseudotime analysis revealed coordinated upregulation of RIPK3, JAK2, STAT1, IRF1, and MLKL, with their expression peaking at a similar pseudotime point (~0.75) (**Figures 4K–P**). Double immunofluorescence further showed increased IRF1, ZBP1, p-STAT1, p-JAK2, p-RIPK3, and p-MLKL in TG-positive follicular cells from HT tissues compared with controls (**Figure 5**).

### Cytokine stimulation induces necroptosis-associated injury signals in thyrocytes

**Figure 6:**
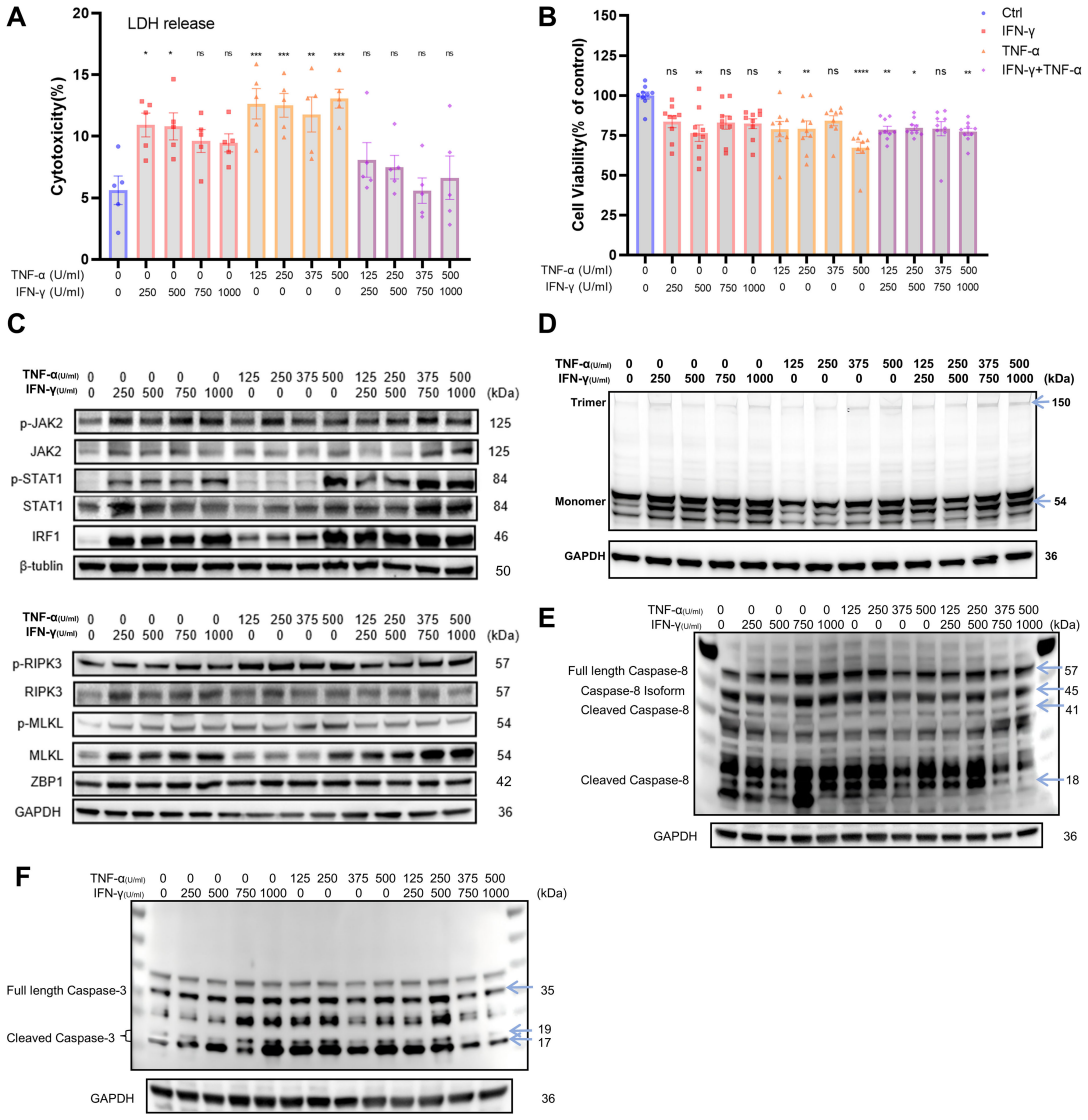
Cytotoxicity, cell viability, and signaling pathway analysis in Nthy-ori 3-1 cells under cytokine stimulation. **(A)** Bar graph illustrating cytotoxicity measured by the Lactate Dehydrogenase (LDH) release assay in Nthy-ori 3-1 cells treated with varying concentrations of TNF-α (0–500 U/mL) and IFN-γ (0–1000 U/mL). **(B)** Bar graph presenting cell viability determined by the CCK-8 assay across the corresponding TNF-α and IFN-γ treatment groups. **(C)** Representative western blot images evaluating the JAK-STAT signaling pathway (p-JAK2, JAK2, p-STAT1, STAT1, IRF1) and necroptosis-related proteins (p-RIPK3, RIPK3, p-MLKL, MLKL, ZBP1) in cell lysates following dose-dependent cytokine treatments. β-tubulin and GAPDH were utilized as internal loading controls. **(D)** Immunoblot analysis of MLKL oligomerization, distinguishing between monomeric (~54 kDa) and trimeric (~150 kDa) forms under the specified cytokine treatment conditions. **(E-F)** Representative western blots analyzing the expression profiles and cleavage statuses of Caspase-8 **(E)** and Caspase-3 **(F)** in response to the varied cytokine treatments. GAPDH served as the loading control. Statistical significance is indicated as follows: *P < 0.05, **P < 0.01, ***P < 0.001, and ****P < 0.0001; ns, not significant.

To model inflammatory injury *in vitro*, Nthy-ori 3-1 cells were treated with TNF-α and IFN-γ. TNF-α induced dose-dependent cytotoxicity, with maximal injury observed at 500 U/mL, whereas IFN-γ did not show an obvious synergistic effect under these conditions (**Figures 6A, B**).

TNF-α stimulation activated the JAK-STAT pathway, as indicated by increased p-JAK2, p-STAT1, and IRF1, and simultaneously induced necroptosis-associated signaling, including p-RIPK3 and MLKL oligomerization (**Figures 6C, D**). Cleavage of caspase-8 and caspase-3 was also detected, indicating concurrent activation of apoptotic signaling (**Figures 6E, F**). In contrast to patient tissues, ZBP1 remained at basal levels in cytokine-stimulated Nthy-ori 3-1 cells (**Figure 6C**).

### Inhibition of downstream cell death pathways attenuates TNF-α-induced thyrocyte injury

**Figure 7:**
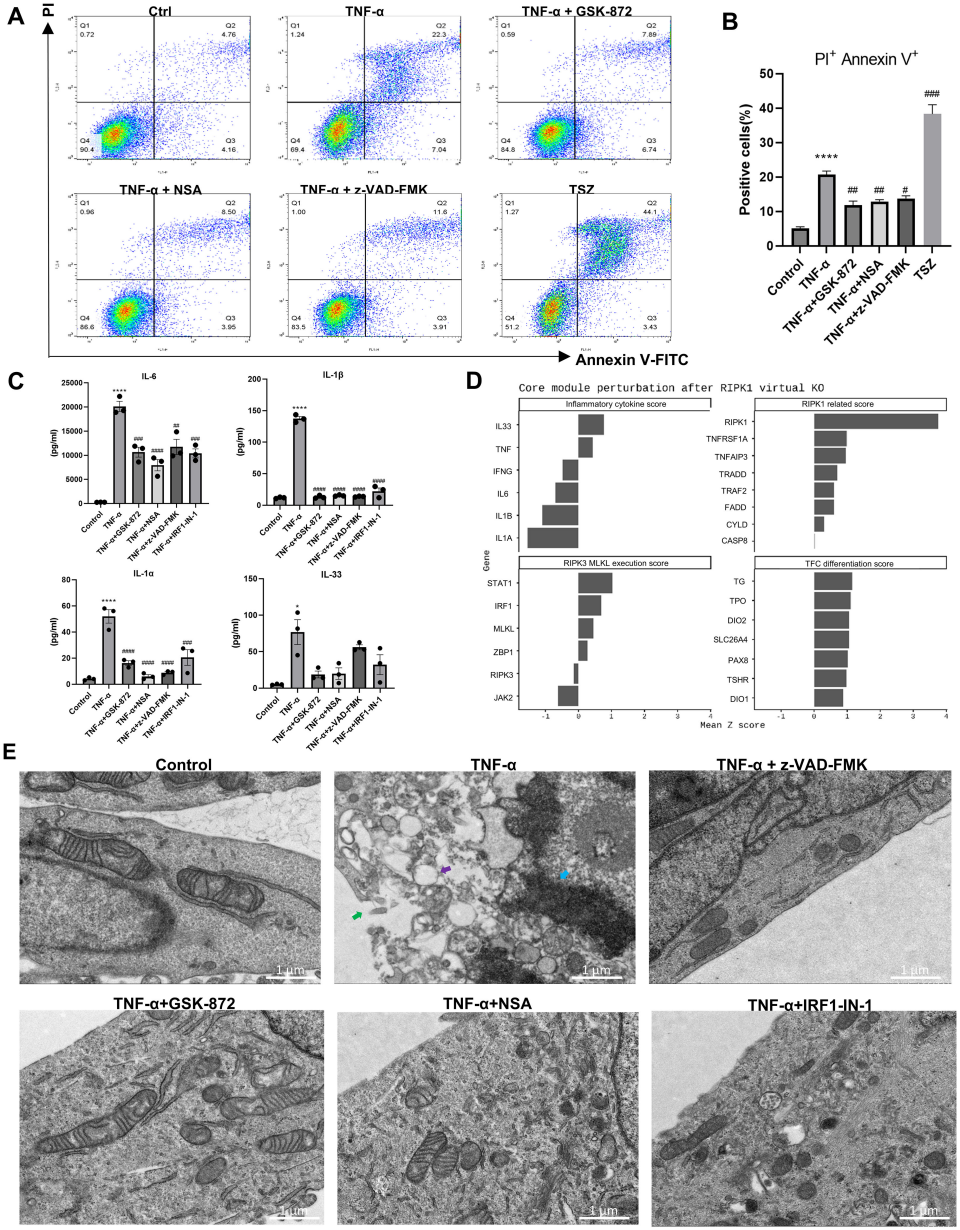
Pharmacological blockade of downstream necroptotic and apoptotic execution pathways protects Nthy-ori 3-1 cells from TNF-α-induced inflammatory injury. **(A)** Representative flow cytometry plots utilizing Annexin V-FITC and Propidium Iodide (PI) double staining. Cells were treated with TNF-α alone, pretreated with specific inhibitors (GSK-872, NSA, z-VAD-FMK), or treated with the TSZ (TNF-α + Smac mimetic + z-VAD-FMK) cocktail. **(B)** Bar graph quantifying the percentage of PI+ and Annexin V+ double-positive cells based on the flow cytometry data acquired in **(A)**. **(C)** Enzyme-linked immunosorbent assay (ELISA) quantification of pro-inflammatory cytokines (IL-6, IL-1β, IL-1α, IL-33) secreted into the culture supernatants across the designated treatment and inhibitor groups, including the IRF1 inhibitor (IRF1-IN-1). **(D)** Exploratory in silico RIPK1 perturbation analysis. Bar plots display the predicted mean Z scores of module genes after computational perturbation of RIPK1, including genes assigned to the inflammatory cytokine, RIPK1 related, RIPK3-MLKL execution, and TFC differentiation modules. Positive and negative values represent the predicted direction of gene-level perturbation effects. **(E)** Transmission electron microscopy (TEM) ultrastructural images of Nthy-ori 3-1 human thyroid follicular epithelial cells subjected to the specified treatments and pharmacological inhibitors. Following treatment with TNF-α (500 U/mL), the cells exhibited necroptotic features, including nuclear chromatin condensation and dispersion (blue arrow), plasma membrane rupture (green arrow), and mitochondrial vacuolization (purple arrow). Scale bars: 1 μm. Asterisks indicate comparisons with the Control group, whereas hash symbols indicate comparisons with the TNF-α group. Statistical significance is indicated as follows: *P or #P < 0.05, **P or ##P < 0.01, ***P or ###P < 0.001, and **P or ####P < 0.0001; ns, not significant.

To determine whether Nthy-ori 3-1 cells possess a functional necroptotic machinery, we first applied the canonical TSZ cocktail consisting of TNF-α, Smac mimetic, and z-VAD-FMK. TSZ treatment induced robust cell death, confirming that these cells are capable of undergoing necroptosis when caspase activity is inhibited (**Figures 7A, B**). In TNF-α-stimulated cells, pharmacological inhibition of RIPK3 with GSK-872, MLKL with NSA, or caspases with z-VAD-FMK significantly reduced Annexin V/PI double-positive cells (**Figures 7A, B**).

Consistently, inhibition of RIPK3, MLKL, or caspases reduced the release of inflammatory cytokines, including IL-6, IL-1β, and IL-1α. IRF1 inhibition also attenuated cytokine secretion under TNF-α stimulation (**Figure 7C**). Transmission electron microscopy further showed that TNF-α-induced ultrastructural injury, including mitochondrial damage and plasma membrane disruption, was partially alleviated by inhibition of RIPK3, MLKL, caspases, or IRF1 (**Figure 7E**). These findings indicate that TNF-α-induced thyrocyte injury in this model involves both necroptotic and apoptotic execution pathways.

To further examine the transcriptional connectivity of RIPK1 in this injury-related program, we performed an exploratory in silico RIPK1 perturbation analysis. The predicted effects on the RIPK3-MLKL execution, inflammatory cytokine, and TFC differentiation modules were limited and heterogeneous (**Figure 7D**; **Supplementary Tables 8**-**10**). Together with the tissue-level observation that total and phosphorylated RIPK1 were not detectably increased in HT samples, this analysis supports a relatively weak transcriptional linkage between RIPK1-associated signals and the RIPK3-MLKL program.

## Discussion

In this study, we provide a molecular characterization of necroptosis-associated thyrocyte injury in Hashimoto’s Thyroiditis. By integrating spatial analysis and functional experiments, we show that the HT microenvironment is associated with inflammatory TFC injury and an RIPK3-MLKL-predominant necroptosis-associated program.

Although total and phosphorylated RIPK1 were not significantly increased in HT tissues, and exploratory virtual perturbation predicted limited RIPK1-associated transcriptional effects, these findings should not be interpreted as evidence of functional RIPK1 independence. Direct inhibition or silencing of RIPK1 would be required to establish whether RIPK1 kinase activity is dispensable in thyrocyte necroptosis. Therefore, our data more conservatively support an RIPK3-MLKL-predominant necroptosis-associated program in HT.While RIPK1 has been implicated as an important regulator of inflammatory and cell-death signaling in diseases such as psoriasis, rheumatoid arthritis, and ulcerative colitis (15), the lack of detectable RIPK1 upregulation in HT tissues is consistent with reports of RIPK3 activation in UVB-induced skin damage and IFN-induced cell death under conditions in which RIPK1 kinase activity may be limited or dispensable (16, 17).

Our findings complement the recent multi-omics study by Mo et al. (18), which identified the hnRNPC-ATF4 axis as a driver of thyrocyte apoptosis and necroptosis via endoplasmic reticulum stress. Rather than delineating functional independence from RIPK1, our work localizes an RIPK3-MLKL-predominant necroptosis-associated program within a vulnerable TFC subpopulation exposed to an inflammatory niche. These observations support the possibility that thyrocyte loss in HT is heterogeneous and governed by multiple molecular checkpoints, but they remain hypothesis-generating. A notable finding is the discrepancy in ZBP1 regulation between patient tissues and *in vitro* models. In HT patients, ZBP1 is upregulated at mRNA level, likely substituting for RIPK1 by directly interacting with RIPK3 via its RHIM domain to initiate necroptosis (17, 19). However, TNF-α/IFN-γ alone failed to induce ZBP1 in Nthy-ori 3-1 cells. This suggests that while IFN signaling is a prerequisite for ZBP1 synthesis (19), its full activation in the HT microenvironment likely requires a “Signal 2”, such as the sensing of Z-nucleic acids or mitochondrial DNA release (20, 21), factors that are absent in standard monolayer cultures. This requirement for specific endogenous ligands explains why thyrocyte death is intrinsically linked to the complex autoimmune niche, highlighting the necessity of organoid models to study HT chronicity.

Our results support a molecular link between necroptosis and HT severity, as the expression levels of ZBP1 and IRF1 strongly correlate with TPOAb and TgAb titers, indicating that the intensity of the necroptotic process parallels the autoimmune response. While the RIPK3-MLKL axis drives cell rupture and the release of IL-1α, IL-1β and IL-6 (16, 22, 23), and these downstream products can serve as accessible circulating surrogates for monitoring active tissue destruction. In contrast, IL-33 behaved differently in this context. These associations require validation in larger prospective cohorts before they can be used for disease monitoring.

Although previous studies identify IL-33 as a key necroptotic DAMP and our *in vitro* data confirm its induction by TNF-α in Nthy-ori 3-1 cells (24), we observed no significant serum IL-33 elevation in HT patients. This suggests that IL-33 functions primarily as a local alarmin within the thyroid microenvironment rather than a systemic marker. Interestingly, while TNF-α treatment also activated apoptotic caspases, blocking necroptosis was sufficient to restore membrane integrity and reduce inflammation. This suggests that under inflammatory conditions, TFCs may exhibit a “mixed” death phenotype (PANoptosis) (5, 25–27), but the necroptotic component is the primary driver of the inflammatory release. Therapeutically, since RIPK1 expression shows no significant difference between HT and normal tissues, our findings suggest that downstream RIPK3/MLKL targeting merits further preclinical evaluation to preserve thyroid function, contrasting with RIPK1-targeting strategies used in other inflammatory conditions (28).

Several limitations should be emphasized. First, the spatial transcriptomic analysis was based on a limited number of sections, including three HT and two control samples in GSE248205. Although the spatial patterns were further examined in independent HT sections from GSE230424, the small number of spatial samples limits statistical generalizability. Second, the TNF-α/IFN-γ *in vitro* stimulation system is a simplified injury model and does not capture infiltrating lymphocytes, macrophages, type I interferons, IL-17, autoantibody-related effects, chronic inflammatory exposure, thyroid tissue architecture, or stromal/immune interactions that contribute to HT. Third, the functional experiments relied on the immortalized Nthy-ori 3-1 cell line; although useful for studying basic thyrocyte injury and cell-death machinery, this model does not fully reproduce primary TFC biology, and the failure to induce ZBP1 *in vitro* indicates that the patient-tissue ZBP1-regulated program was not recapitulated. Fourth, no direct pharmacological inhibition or genetic silencing of RIPK1 was performed, so our data do not establish functional RIPK1 independence. Fifth, because no *in vivo* HT model was included, the protective effects of RIPK3 or MLKL inhibition observed *in vitro* cannot be directly extrapolated to disease modification *in vivo*. Accordingly, downstream RIPK3-MLKL targeting should be viewed as an experimental therapeutic hypothesis and a potential preclinical target requiring further validation in primary thyrocytes, organoids, immune-thyrocyte co-cultures, and *in vivo* HT models.

## Data Availability

The datasets analyzed during the current study are available in public repositories. The transcriptomic datasets can be accessed via the Gene Expression Omnibus (GEO) under accession numbers GSE248205, GSE230424, GSE29315 and GSE138198, and via the National Genomics Data Center under accession number HRA001684.
