# Supplementary material for: Spatial transcriptomic analysis identifies a necroptosis-prone thyroid follicular cell state in Hashimoto’s thyroiditis
Source: Front Immunol. 2026 Aug 3;17:1902510. doi: 10.3389/fimmu.2026.1902510 (PMC13478095; doi:10.3389/fimmu.2026.1902510)
Supplement: Supplementary file 1 [file Table1.docx]

**Supplementary Methods**

**Supplementary methods for clinical cohorts and cell culture**

Patients in the HT group met the following criteria: age 18–70 years, positive serum antibodies (TgAb ≥ 50 IU/mL or TPOAb ≥ 200 IU/mL), postoperative pathological confirmation of HT, and euthyroid status. Control subjects were euthyroid individuals aged 18–70 years with negative TPOAb and TgAb and no pathological evidence of HT. Exclusion criteria for both groups included coexisting autoimmune disease, radioactive iodine therapy within the previous year, severe systemic chronic disease, active infection, and use of thyroid hormone replacement or anti-thyroid medication. Demographic and clinical characteristics are summarized in Supplementary Tables 13 and 14. The normal reference ranges were FT3 2.43–6.01 pmol/L, FT4 9.01–19.05 pmol/L, TSH 0.35–4.94 mIU/L, TPOAb 0.00–5.61 IU/mL, and TgAb 0.00–4.11 IU/mL.

Nthy-ori 3-1 cells were cultured in RPMI-1640 supplemented with 10% fetal bovine serum, 1% sodium pyruvate, and non-essential amino acids at 37°C in 5% CO2. To simulate the inflammatory microenvironment, cells were treated with recombinant human TNF-α and IFN-γ. For inhibition experiments, cells were pretreated with GSK-872, necrosulfonamide, z-VAD-FMK, or IRF1-IN-1 before cytokine stimulation.

**Supplementary methods for histology, immunostaining, and TEM**

Formalin-fixed paraffin-embedded thyroid tissues were sectioned at 4 μm. Hematoxylin and eosin staining was performed using standard procedures. For immunohistochemistry, antigen retrieval was carried out in EDTA buffer, followed by incubation with primary antibodies overnight at 4°C and visualization using a biotin–streptavidin–HRP/DAB system. For immunofluorescence, tissue sections were co-stained with thyroglobulin and target proteins, followed by fluorescence-conjugated secondary antibodies and DAPI nuclear counterstaining. Detailed antibody information is listed in Supplementary Table 15.

For ultrastructural analysis, fresh thyroid tissues or cell pellets were fixed in electron microscopy fixative, post-fixed in 1% osmium tetroxide, dehydrated, and embedded in resin. Ultrathin sections (60–80 nm) were stained with uranyl acetate and lead citrate and examined by transmission electron microscopy to assess necroptotic changes such as organelle swelling, vacuolization, and plasma membrane disruption.

**Supplementary methods for molecular and functional assays**

Total RNA was extracted and reverse-transcribed into cDNA for quantitative real-time PCR analysis. Primer sequences are listed in Supplementary Table 16. For protein analysis, cell or tissue lysates were prepared in RIPA buffer, quantified, separated by standard or non-reducing SDS-PAGE, and analyzed by immunoblotting. Cytokine levels in serum and cell culture supernatants were measured by ELISA according to the manufacturers’ instructions. Serum samples were diluted 1:100 before analysis. Cell viability and death were evaluated using CCK-8, LDH release, and Annexin V-EGFP/PI flow cytometry assays.
