# Supplementary material for: Spatial transcriptomic analysis identifies a necroptosis-prone thyroid follicular cell state in Hashimoto’s thyroiditis
Source: Front Immunol. 2026 Aug 3;17:1902510. doi: 10.3389/fimmu.2026.1902510 (PMC13478095; doi:10.3389/fimmu.2026.1902510)
Supplement: Supplementary file 2 [file Table2.docx]

Supplementary Table 1. Summary of sequencing quality control metrics and spatial spot counts for each sample.

| **Sample** | **Valid Spots** | **Mean Genes** | **Median Genes** | **Mean UMI** | **Median UMI** | **Mean MT** | **Median MT** |
| --- | --- | --- | --- | --- | --- | --- | --- |
| C1 | 1031 | 956 | 819 | 1845 | 1382 | 9.05 | 8.9 |
| C2 | 1646 | 765 | 716 | 1333 | 1174 | 11.74 | 11.47 |
| HT1 | 1911 | 1737 | 1669 | 6055 | 5234 | 2.18 | 1.63 |
| HT2 | 2591 | 1704 | 1612 | 6464 | 5489 | 3.91 | 3.07 |
| HT3 | 1979 | 2465 | 2524 | 7835 | 7208 | 3.38 | 2.91 |

The table displays quality-control (QC) metrics for the five spatial transcriptomic sections analyzed. Samples C1 and C2 represent controls, whereas HT1, HT2, and HT3 represent Hashimoto’s thyroiditis (HT) sections. Metrics include the number of valid tissue-covered spots, genes detected per spot, UMIs per spot, and mitochondrial gene percentage.

Supplementary Table 2. Spatial cluster annotation and representative lineage-enriched marker genes in the Control group.

| **Annotated Spatial Cluster** | **Marker Genes** |
| --- | --- |
| TFC2 | TPO, PAX8, KLF6, SRF, ERRFI1, ATF3, EGR2, NR4A1 |
| TFC3 | TPO, PAX8, PCP4, MRPS21, CHCHD10, EIF5A, NPNT |
| Fibroblast | SFRP2, IGKC, C1R, FBLN1, DCN |
| TFC1 | TPO, PAX8, PLCG2, AL627171.2, HES1, MLEC, IGLC2, PTCH2 |
| Immune Cell | HBB, HBA2, PLCG2, IGLC2, IGKC |
| Endothelial | DES, ACTG2, MYH11, ACTA2, TPM2 |
| SMC | NTS, ACKR1, APLNR, TGM2, SFRP4 |

Clustering was performed using the top 30 principal components (PCs) based on the ElbowPlot heuristic. Seven distinct clusters were identified and annotated into specific cell types based on the expression of canonical marker genes. Abbreviations: TFC, Thyroid Follicular Cell.

Supplementary Table 3. Spatial cluster annotation and representative lineage-enriched marker genes in the Hashimoto’s thyroiditis (HT) group.

| **Annotated Spatial Cluster** | **Marker Genes** |
| --- | --- |
| T cell | CCL18, CD69, PASK, LINC01781, SELL |
| Fibroblast | DES, CCN5, PI16, HBA2, PPP1R14A |
| TFC1 | TPO, PAX8, MT1H, MT1G, CLIC6, ARG2, C11orf74 |
| Plasma | MALAT1, IYD, DAPL1, IGLC1, IGKC |
| Endothelial | CXCL14, RPL17, CD99, CCDC80, ALOX5AP |
| Macrophage | SPP1, C15orf48, CXCL11, CHI3L1, CXCL10 |
| TFC2 | TPO, PAX8, FNDC1, SFTA3, CLDN4, KRT19, SLC22A17 |
| B cell | FDCSP, CR2, ELL3, RGS13, LTF |
| TFC3 | TPO, PAX8, TCERG1L, AC253572.2, AC007952.4, SMOC2, DUOXA2 |

Similar to the control group, cells from the HT samples were clustered and annotated. Nine clusters were defined, revealing disease-specific heterogeneity including distinct immune cell populations (e.g., T cells, B cells, Plasma cells, Macrophages) and stromal cells (Fibroblast). Markers listed are the top differentially expressed genes used for annotation.

Supplementary Table 4. Distribution and proportion of annotated spatial clusters across Control and HT sections.

| Group | Cell Type | Spot Count | Percentage of Spots |
| --- | --- | --- | --- |
| Control | TFC2 | 903 | 33.731789316399 |
| Control | TFC1 | 780 | 29.1370937616735 |
| Control | TFC3 | 324 | 12.1031004856182 |
| Control | Fibroblast | 291 | 10.870377288009 |
| Control | Immune Cell | 228 | 8.51699663802764 |
| Control | Endothelial | 118 | 4.40791931266343 |
| Control | SMC | 33 | 1.23272319760926 |
| HT | T cells | 1166 | 17.991050763771 |
| HT | Fibroblast | 1106 | 17.065267705601 |
| HT | TFC1 | 1002 | 15.4605770714396 |
| HT | Plasma | 886 | 13.6707298256442 |
| HT | Endothelial | 666 | 10.2761919456874 |
| HT | Macrophage | 575 | 8.87208764079617 |
| HT | TFC2 | 530 | 8.17775034716865 |
| HT | B cells | 419 | 6.46505168955408 |
| HT | TFC3 | 131 | 2.02129301033791 |

The table compares the spatial cluster composition between Control and HT sections. Data represent the number of spots assigned to each annotated spatial cluster and the percentage relative to the total number of valid spots within each group.

Supplementary Table 5. Aggregated ligand–receptor communication adjacency matrix among annotated spatial clusters in Hashimoto’s thyroiditis.

| Source | B cell | Endothelial | Fibroblast | Macrophage | Plasma | T cell | TFC1 | TFC2 | TFC3 |
| --- | --- | --- | --- | --- | --- | --- | --- | --- | --- |
| B cell | 1.53297 76 | 0.00 0 | 0.2230325 19 | 0.8675354 77 | 0.0736993 7 | 1.435137 64 | 0.1851252 31 | 0.4372738 43 | 0.4413822 62 |
| Endothelial | 0.5476064 13 | 0.00 0 | 0.0112518 1 | 0.2399684 8 | 0.00 0 | 0.7555089 18 | 0.00 0 | 0.1409908 1 | 0.01235499 1 |
| Fibroblast | 1.373276 52 | 0.00 0 | 0.3036629 15 | 1.097085 61 | 0.07410871 12 | 1.351961 45 | 0.1891314 27 | 0.4566238 33 | 0.850957 46 |
| Macrophage | 1.658508 88 | 0.09865605 1 | 0.3130599 24 | 1.166727 96 | 0.1638466 19 | 1.582895 70 | 0.2859487 47 | 0.5848833 60 | 0.9310934 95 |
| Plasma | 1.026328 31 | 0.00 0 | 0.03273512 3 | 0.6015387 33 | 0.00 0 | 1.083824 30 | 0.02552582 6 | 0.2091217 9 | 0.3244291 19 |
| T cell | 1.335491 67 | 0.00 0 | 0.1827743 14 | 0.7749997 61 | 0.06148558 2 | 1.333047 55 | 0.1098424 18 | 0.370325 36 | 0.2638307 49 |
| TFC1 | 1.261095 50 | 0.09902345 1 | 0.2869034 11 | 1.034038 59 | 0.1225538 7 | 1.306943 45 | 0.2095044 24 | 0.460184 29 | 0.6539612 47 |
| TFC2 | 1.543513 73 | 0.1006627 1 | 0.2144832 17 | 1.262062 83 | 0.1790291 18 | 1.491917 59 | 0.3163906 41 | 0.5751531 49 | 0.9643373 73 |
| TFC3 | 1.575796 75 | 0.2246755 1 | 0.3458099 21 | 1.166347 97 | 0.322257 24 | 1.525625 59 | 0.5387743 59 | 0.8126713 62 | 1.466358 110 |

This table presents the aggregated interaction weights inferred by CellChat analysis, where rows represent ligand-enriched sender clusters (Source) and columns represent receptor-enriched receiver clusters (Target); the values indicate the sum of communication probabilities for all significant ligand-receptor pairs detected between each cell pair, with higher values indicate stronger inferred ligand–receptor communication strength between annotated spatial clusters. Abbreviation: TFC, Thyroid Follicular Cells.

Supplementary Table 6. Aggregated inferred signaling strengths of annotated spatial clusters in the Hashimoto’s thyroiditis microenvironment.

| **Annotated Spatial Cluster** | **Outgoing** | **Incoming** |
| --- | --- | --- |
| B cells | 5.19615500796505 | 11.854582281837 |
| Endothelial | 1.70768130847612 | 0.523017703227154 |
| Fibroblast | 5.69680650688094 | 1.9137129311745 |
| Macrophage | 6.78561885520305 | 8.21030098323921 |
| Plasma | 3.30350162813614 | 0.996980075383447 |
| T cells | 4.43179551780378 | 11.8668572420399 |
| TFC1 | 5.43420521191397 | 1.86024284218302 |
| TFC2 | 6.64754754161282 | 4.04722699408136 |
| TFC3 | 7.97831305649954 | 5.90870358132586 |

This table summarizes the total outgoing and incoming interaction strengths for each spatial cluster, derived from the inferred intercellular communication network (CellChat). Outgoing strength reflects the sum of communication probabilities for all signaling pathways in which a spatial cluster acts as a sender, indicating its capacity to drive intercellular signaling, while incoming strength represents the sum of communication probabilities where the cell type acts as a receiver, indicating its susceptibility to microenvironmental signals. Abbreviation: TFC, Thyroid Follicular Cells.

Supplementary Table 7. Spot-level spatial correlations among predefined module scores in HT spatial transcriptomic sections.

| Dataset | Sample | Score X | Score Y | Spearman rho | P value | N spots |
| --- | --- | --- | --- | --- | --- | --- |
| GSE248205 | HT1 | RIPK3 MLKL execution score | Inflammatory cytokine score | 0.113851248 | 2.50761E-07 | 2041 |
| GSE248205 | HT1 | RIPK3 MLKL execution score | TFC differentiation score | 0.072998438 | 0.000965936 | 2041 |
| GSE248205 | HT1 | RIPK3 MLKL execution score | RIPK1 related score | 0.013938073 | 0.529132326 | 2041 |
| GSE248205 | HT1 | RIPK1 related score | Inflammatory cytokine score | 0.057526872 | 0.009336599 | 2041 |
| GSE248205 | HT2 | RIPK3 MLKL execution score | Inflammatory cytokine score | 0.231126607 | 3.60074E-33 | 2625 |
| GSE248205 | HT2 | RIPK3 MLKL execution score | TFC differentiation score | -0.072208021 | 0.000213374 | 2625 |
| GSE248205 | HT2 | RIPK3 MLKL execution score | RIPK1 related score | 0.064827712 | 0.000889445 | 2625 |
| GSE248205 | HT2 | RIPK1 related score | Inflammatory cytokine score | 0.099530013 | 3.22742E-07 | 2625 |
| GSE248205 | HT3 | RIPK3 MLKL execution score | Inflammatory cytokine score | 0.196775533 | 3.71169E-20 | 2144 |
| GSE248205 | HT3 | RIPK3 MLKL execution score | TFC differentiation score | -0.220007487 | 6.50482E-25 | 2144 |
| GSE248205 | HT3 | RIPK3 MLKL execution score | RIPK1 related score | 0.052433052 | 0.015178987 | 2144 |
| GSE248205 | HT3 | RIPK1 related score | Inflammatory cytokine score | 0.116564732 | 6.20989E-08 | 2144 |
| GSE230424 | P3 | RIPK3 MLKL execution score | Inflammatory cytokine score | 0.153198254 | 9.33405E-23 | 4062 |
| GSE230424 | P3 | RIPK3 MLKL execution score | TFC differentiation score | -0.106337986 | 1.0868E-11 | 4062 |
| GSE230424 | P3 | RIPK3 MLKL execution score | RIPK1 related score | 0.075670125 | 1.37783E-06 | 4062 |
| GSE230424 | P3 | RIPK1 related score | Inflammatory cytokine score | 0.050174067 | 0.001379934 | 4062 |
| GSE230424 | P4 | RIPK3 MLKL execution score | Inflammatory cytokine score | 0.22500458 | 3.54378E-54 | 4626 |
| GSE230424 | P4 | RIPK3 MLKL execution score | TFC differentiation score | -0.204964584 | 4.58866E-45 | 4626 |
| GSE230424 | P4 | RIPK3 MLKL execution score | RIPK1 related score | 0.036971591 | 0.011910263 | 4626 |
| GSE230424 | P4 | RIPK1 related score | Inflammatory cytokine score | 0.050481262 | 0.000593144 | 4626 |

Spearman correlation analysis was performed at the spot level in HT sections from two spatial transcriptomic datasets, GSE248205 and GSE230424. The table summarizes the correlations between the RIPK3-MLKL execution score and inflammatory cytokine score, TFC differentiation score, and RIPK1 related score, as well as the correlation between the RIPK1 related score and inflammatory cytokine score.

Supplementary Table 8. Composition and abundance of major cell populations in the HRA001684 single-cell thyroid dataset.

| Cell type | Cell number | Cell fraction | |
| --- | --- | --- | --- |
| T cell | 11103 | 0.183706 |  |
| B cell | 10748 | 0.177832 |  |
| Plasma cell | 9633 | 0.159384 |  |
| Endothelial | 7582 | 0.125449 |  |
| NK T cell | 5879 | 0.097272 | |
| SMC Pericyte | 4486 | 0.074224 | |
| Macrophage | 3895 | 0.064445 | |
| Proliferating | 3069 | 0.050778 | |
| Fibroblast | 2797 | 0.046278 | |
| TFC | 937 | 0.015503 | |
| Lymphatic Endothelial | 310 | 0.005129 | |

The table shows the absolute cell numbers across 11 annotated cell populations in the HRA001684 single-cell transcriptomic dataset. TFCs comprised 937 cells and were prioritized for downstream virtual knockout analysis.

Supplementary Table 9. Top genes predicted to be perturbed by RIPK1 virtual knockout in TFCs.

| Gene | Mean distance | Mean Z | Median Z | Mean FC | Median FC | Median p adj | Significant seed number | Total seed number | |
| --- | --- | --- | --- | --- | --- | --- | --- | --- | --- |
| RIPK1 | 1.053961e-05 | 3.753177 | 4.075935 | 276007.141180 | 253177.324998 | 0.000000 | 3 | 3 |  |
| CXCL12 | 7.263935e-08 | 1.393160 | 1.327984 | 14.731379 | 12.178060 | 0.026971 | 3 | 3 |  |
| DCN | 1.114979e-07 | 1.407432 | 1.481252 | 22.901087 | 31.290208 | 0.000033 | 2 | 3 |  |
| COL1A2 | 1.085932e-07 | 1.391820 | 1.472183 | 21.643296 | 29.626405 | 0.000049 | 2 | 3 |  |
| LUM | 1.069168e-07 | 1.384949 | 1.469916 | 21.005530 | 29.223871 | 0.000049 | 2 | 3 | |
| COLEC12 | 1.048952e-07 | 1.379352 | 1.461683 | 20.209320 | 27.805255 | 0.000067 | 2 | 3 | |
| COL6A3 | 1.038969e-07 | 1.373772 | 1.462925 | 19.861987 | 28.014997 | 0.000067 | 2 | 3 | |
| DPT | 1.018211e-07 | 1.364288 | 1.453535 | 19.019539 | 26.465901 | 0.000115 | 2 | 3 | |
| VCAN | 9.891428e-08 | 1.349206 | 1.450802 | 17.996297 | 25.282779 | 0.000127 | 2 | 3 | |
| SERPINF1 | 1.002421e-07 | 1.361964 | 1.444621 | 18.423677 | 25.070975 | 0.000166 | 2 | 3 | |
| CHRDL1 | 9.922783e-08 | 1.357583 | 1.443139 | 18.066713 | 24.845845 | 0.000170 | 2 | 3 | |
| LHFPL6 | 1.070372e-07 | 1.410133 | 1.441928 | 21.158359 | 24.663494 | 0.000171 | 2 | 3 | |
| CFH | 9.563031e-08 | 1.373663 | 1.429678 | 17.150522 | 22.888084 | 0.000369 | 2 | 3 | |
| COL3A1 | 9.282162e-08 | 1.337441 | 1.424425 | 15.879414 | 22.164550 | 0.000503 | 2 | 3 | |
| OGN | 9.181102e-08 | 1.338839 | 1.422473 | 15.606906 | 21.855394 | 0.000540 | 2 | 3 | |
| SFRP2 | 9.365893e-08 | 1.334973 | 1.424961 | 16.399256 | 21.007274 | 0.000660 | 2 | 3 | |
| PRRX1 | 9.397919e-08 | 1.346371 | 1.417974 | 16.220820 | 21.305804 | 0.000694 | 2 | 3 | |
| FBLN1 | 9.078920e-08 | 1.318013 | 1.411686 | 15.045100 | 20.499322 | 0.000998 | 2 | 3 | |
| CCDC80 | 9.144945e-08 | 1.331146 | 1.410857 | 15.322054 | 20.395269 | 0.000998 | 2 | 3 | |
| FXYD1 | 9.681797e-08 | 1.388260 | 1.408176 | 17.593246 | 20.061844 | 0.001027 | 2 | 3 | |
| C7 | 9.080242e-08 | 1.332078 | 1.412345 | 15.478953 | 19.664288 | 0.001209 | 2 | 3 | |
| OLFML2B | 8.585120e-08 | 1.311549 | 1.400291 | 13.705198 | 18.456943 | 0.002013 | 2 | 3 | |
| ABCA8 | 8.396221e-08 | 1.286681 | 1.397285 | 12.957753 | 18.166873 | 0.002258 | 2 | 3 | |
| SRPX | 8.902237e-08 | 1.397879 | 1.393463 | 16.085465 | 18.322747 | 0.002340 | 2 | 3 | |
| ABCA6 | 8.313752e-08 | 1.298905 | 1.390719 | 12.766246 | 17.949289 | 0.002641 | 2 | 3 | |
| SVEP1 | 8.346188e-08 | 1.281346 | 1.384234 | 12.930926 | 16.956753 | 0.003839 | 2 | 3 | |
| SERPINE2 | 7.837518e-08 | 1.285419 | 1.368401 | 11.434171 | 15.590363 | 0.006581 | 2 | 3 | |
| MGP | 7.820282e-08 | 1.312006 | 1.366501 | 11.633911 | 15.501375 | 0.007445 | 2 | 3 | |
| TNC | 7.984858e-08 | 1.277044 | 1.368201 | 11.652476 | 15.666277 | 0.007445 | 2 | 3 | |
| SPARCL1 | 6.946387e-08 | 1.369734 | 1.363401 | 13.528129 | 15.180902 | 0.007743 | 2 | 3 | |

The table summarizes the top genes predicted to be perturbed after in silico RIPK1 virtual knockout in TFCs. Perturbation effects were quantified by mean Z-scores across three independent random seeds, reflecting predicted structural divergence in the inferred gene regulatory network.

Supplementary Table 10. Predicted impact of RIPK1 virtual knockout on core necroptosis-, inflammation-, and differentiation-related genes.

| **Gene** | **Mean distance** | **Mean Z** | **Median Z** | **Mean FC** | **Median FC** | **Median p adj** | **Significant seed number** | **Total seed number** |
| --- | --- | --- | --- | --- | --- | --- | --- | --- |
| IL33 | 1.634944e-08 | 0.777471 | 0.796780 | 5.226320e-01 | 5.462127e-01 | 1.000000 | 0 | 3 |
| TNF | 5.145597e-09 | 0.423340 | 0.540180 | 4.751761e-02 | 4.184569e-02 | 1.000000 | 0 | 3 |
| IFNG | 5.764487e-10 | -0.489438 | -0.796651 | 1.667214e-03 | 6.465660e-08 | 1.000000 | 0 | 3 |
| IL6 | 1.085581e-10 | -0.700997 | -0.711860 | 1.425361e-03 | 2.505173e-07 | 1.000000 | 0 | 3 |
| IL1B | 9.978775e-13 | -1.086508 | -0.882204 | 7.915467e-08 | 8.111076e-10 | 1.000000 | 0 | 3 |
| IL1A | 1.906529e-16 | -1.540745 | -1.519338 | 4.064904e-15 | 2.022843e-18 | 1.000000 | 0 | 3 |
| RIPK1 | 1.053961e-05 | 3.753177 | 4.075935 | 2.760071e+05 | 2.531773e+05 | 0.000000 | 3 | 3 |
| TNFAIP3 | 3.616503e-08 | 0.965248 | 1.104405 | 2.335094e+00 | 2.828263e+00 | 0.943209 | 0 | 3 |
| TNFRSF1A | 2.412617e-08 | 0.988784 | 1.023774 | 1.422865e+00 | 1.444251e+00 | 1.000000 | 0 | 3 |
| TRADD | 9.883365e-09 | 0.708162 | 0.698170 | 2.344055e-01 | 2.098821e-01 | 1.000000 | 0 | 3 |
| TRAF2 | 6.482308e-09 | 0.609494 | 0.632382 | 1.134689e-01 | 1.006658e-01 | 1.000000 | 0 | 3 |
| FADD | 7.889561e-09 | 0.603638 | 0.574890 | 1.285205e-01 | 1.214589e-01 | 1.000000 | 0 | 3 |
| CYLD | 2.168860e-09 | 0.297303 | 0.348048 | 2.309589e-02 | 2.243934e-02 | 1.000000 | 0 | 3 |
| CASP8 | 4.454169e-10 | 0.014910 | 0.046610 | 1.720790e-03 | 4.173496e-04 | 1.000000 | 0 | 3 |
| STAT1 | 2.867986e-08 | 1.032041 | 1.041829 | 1.856759e+00 | 1.853197e+00 | 1.000000 | 0 | 3 |
| IRF1 | 1.417601e-08 | 0.689157 | 0.813430 | 3.594784e-01 | 4.388045e-01 | 1.000000 | 0 | 3 |
| MLKL | 2.702883e-09 | 0.446777 | 0.219637 | 2.494933e-01 | 7.627869e-03 | 1.000000 | 0 | 3 |
| ZBP1 | 2.984277e-09 | 0.272813 | 0.177325 | 3.386950e-02 | 5.437824e-03 | 1.000000 | 0 | 3 |
| RIPK3 | 8.358771e-10 | -0.146336 | 0.148537 | 3.180857e-03 | 4.358482e-03 | 1.000000 | 0 | 3 |
| JAK2 | 2.178094e-11 | -0.625121 | -0.749059 | 5.282449e-05 | 1.076897e-08 | 1.000000 | 0 | 3 |
| TG | 3.756050e-08 | 1.152011 | 1.179809 | 3.940662e+00 | 4.682471e+00 | 0.816586 | 0 | 3 |
| TPO | 3.277669e-08 | 1.106538 | 1.106538 | 2.901486e+00 | 2.869392e+00 | 1.000000 | 0 | 3 |
| DIO2 | 2.956524e-08 | 1.056394 | 1.100886 | 2.190197e+00 | 2.230072e+00 | 1.000000 | 0 | 3 |
| SLC26A4 | 2.939523e-08 | 1.055121 | 1.104745 | 2.218385e+00 | 2.256774e+00 | 1.000000 | 0 | 3 |
| PAX8 | 2.584934e-08 | 1.022613 | 1.042241 | 1.747147e+00 | 1.848620e+00 | 1.000000 | 0 | 3 |
| TSHR | 2.251405e-08 | 0.985290 | 0.995702 | 1.392743e+00 | 1.336677e+00 | 1.000000 | 0 | 3 |
| DIO1 | 1.718655e-08 | 0.884318 | 0.949917 | 9.123647e-01 | 9.508213e-01 | 1.000000 | 0 | 3 |

This table reports perturbation metrics for predefined genes related to inflammatory cytokines, RIPK1-associated signaling, RIPK3-MLKL execution, and thyroid differentiation after in silico RIPK1 virtual knockout.

Supplementary Table 11. Differentially expressed genes (DEGs) between Hashimoto's Thyroiditis and normal control tissues in the GSE138198 dataset.

Supplementary Table 12. Differentially expressed genes (DEGs) between Hashimoto's Thyroiditis and normal control tissues in the HRA001684 dataset.

Supplementary Table 13. Demographic and clinical characteristics of study participants with thyroid tissue samples.

|  | Control (N=30) | HT(N=26) | *P*-value |
| --- | --- | --- | --- |
| Age (years) | 43.87±10.30 | 45.69±14.10 | 0.5791 |
| Sex（Female/male） | 18/12 | 22/4 | 0.0737 |
| FT3(pmol/L) | 4.63±0.44 | 4.55±0.42 | 0.4551 |
| FT4(pmol/L) | 13.03±1.13 | 13.18±1.64 | 0.7291 |
| TSH(mIU/L) | 1.77±0.79 | 1.98±0.99 | 0.3150 |
| TPOAb(IU/mL) | 0.74(0.45-1.39) | 405.1(38.78-992.1) | <0.0001 |
| TgAb(IU/mL) | 1.19(0.71-1.79) | 172.4(96.16-589.9) | <0.0001 |

The normal reference ranges were obtained from the manufacturer (Abbott): free T3 (FT3): 2.63–5.70 pmol/l; free T4 (FT4): 9.01–19.05 pmol/l; thyrotropin (TSH): 0.35–4.94 mIU/l; thyroid peroxidase antibody (TPOAb): 0.00–5.61 IU/ml; thyroglobulin antibody (TgAb): 0.00–4.11 IU/ml.

Supplementary Table 14. Demographic and clinical characteristics of study participants with serum samples.

|  | Control (N=17) | HT (N=17) | *P*-value |
| --- | --- | --- | --- |
| Age (years) | 48.35±16.01 | 51.76±12.47 | 0.5834 |
| Sex（Female/male） | 13/4 | 15/2 | 0.6562 |
| FT3(pmol/L) | 4.60±0.39 | 4.52±0.72 | 0.4184 |
| FT4(pmol/L) | 12.56±1.01 | 13.82±1.87 | 0.0386 |
| TSH(mIU/L) | 1.46±0.84 | 2.06±1.30 | 0.1598 |
| TPOAb(IU/mL) | 0.55(0.025-0.95) | 607.5(117.1-1000) | <0.0001 |
| TgAb(IU/mL) | 1.24(0.90-1.48) | 291(36.28-485.8) | <0.0001 |

The normal reference ranges were obtained from the manufacturer (Abbott): free T3 (FT3): 2.63–5.70 pmol/l; free T4 (FT4): 9.01–19.05 pmol/l; thyrotropin (TSH): 0.35–4.94 mIU/l; thyroid peroxidase antibody (TPOAb): 0.00–5.61 IU/ml; thyroglobulin antibody (TgAb): 0.00–4.11 IU/ml.

Supplementary Table 15. List of primary and secondary antibodies used for Western blotting.

| Antibody | Dilution | Cat. No. | Source |
| --- | --- | --- | --- |
| p-RIPK1 | 1:1000 | #AF2398 | Affinity Biosciences, Liyang, China |
| RIPK1 | 1:1000 | #A7414 | Abclonal, Wuhan, China |
| p-JAK2 | 1:500 | #AP0531 | Abclonal, Wuhan, China |
| JAK2 | 1:1000 | #BM4165 | BOSTER, CA, USA |
| p-STAT1 | 1:1000 | #AF3300 | Affinity Biosciences, Liyang, China |
| STAT1 | 1:5000 | #66545-1-Ig | Proteintech, Wuhan, China |
| p-RIPK3 | 1:2000 | #ab209384 | Abcam, Cambridge, UK |
| RIPK3 | 1:1000 | #17563-1-AP | Proteintech, Wuhan, China |
| p-MLKL | 1:1000 | #ab187091 | Abcam, Cambridge, UK |
| MLKL | 1:1000 | #ab184718 | Abcam, Cambridge, UK |
| ZBP1 | 1:1000 | #13285-1-AP | Proteintech, Wuhan, China |
| IRF1 | 1:1000 | #8478 | Cell Signaling Technology, MA, USA |
| Caspase 3 | 1:1000 | #19677-1-AP | Proteintech, Wuhan, China |
| Caspase 8 | 1:2000 | #66093-1-Ig | Proteintech, Wuhan, China |
| Thyroglobulin | 1:500 | #GB14156-50 | Servicebio, Wuhan, China |
| GAPDH | 1:2000 | #60004-1-Ig | Proteintech, Wuhan, China |
| Beta Tubulin | 1:2000 | #10094-1-AP | Proteintech, Wuhan, China |
| HRP-conjugated goat anti-rabbit IgG | 1:5000 | #WLA023 | Wanleibio, Shenyang, China |
| HRP-conjugated rabbit anti-mouse IgG | 1:5000 | #WLA024 | Wanleibio, Shenyang, China |

Abbreviations: Cat. No., catalog number; HRP, horseradish peroxidase; p-, phosphorylated. The dilution ratios indicate the working concentration used in the experiments.

Supplementary Table 16. Primer sequences used for quantitative real-time PCR.

| **Gene** | **Sequence** |
| --- | --- |
| GAPDH-F | GCACCGTCAAGGCTGAGAAC |
| GAPDH-R | TGGTGAAGACGCCAGTGGA |
| RIPK3-F | ATGTCGTGCGTCAAGTTATGG |
| RIPK3-R | CGTAGCCCCACTTCCTATGTTG |
| MLKL-F | AGGAGGCTAATGGGGAGATAGA |
| MLKL-R | TGGCTTGCTGTTAGAAACCTG |
| JAK2-F | ATCCACCCAACCATGTCTTCC |
| JAK2-R | ATTCCATGCCGATAGGCTCTG |
| STAT1-F | CAGCTTGACTCAAAATTCCTGGA |
| STAT1-R | TGAAGATTACGCTTGCTTTTCCT |
| IRF1-F | CTGTGCGAGTGTACCGGATG |
| IRF1-R | ATCCCCACATGACTTCCTCTT |
| ZBP1-F | AACATGCAGCTACAATTCCAGA |
| ZBP1-R | AGTCTCGGTTCACATCTTTTGC |
| IFNG-F | TCGGTAACTGACTTGAATGTCCA |
| IFNG-R | TCGCTTCCCTGTTTTAGCTGC |
| TNFA-F | CCTCTCTCTAATCAGCCCTCTG |
| TNFA-R | GAGGACCTGGGAGTAGATGAG |

F, forward primer; R, reverse primer. All sequences are listed in the 5' to 3' direction.

Supplementary Figure 1. Quality-control metrics and spatial cluster composition mapping for spatial transcriptomic data.

(A, D) Violin plots displaying the distribution of detected gene counts (nFeature_RNA), total unique molecular identifiers (nCount_RNA), and the percentage of mitochondrial gene expression (percent.mt) per spot for individual Control (A) and HT (D) samples prior to downstream analysis.

(B, E) Scatter plots illustrating the correlation between UMI counts per spot and Gene counts per spot, color-coded by the mitochondrial gene percentage, for Control (B) and HT (E) datasets.

(C, F) Stacked bar charts (left) and pie charts (right) presenting the proportional distribution of annotated spatial clusters within individual tissue sections and across the merged Control (C) and HT (F) cohorts.

Supplementary Figure 2. Expression profiling of canonical marker genes for cell-type-enriched spatial cluster annotation.

(A-B) Dot plots illustrating the expression patterns of established lineage and subpopulation marker genes across the distinct cell clusters identified in the scRNA-seq analysis. The size of each dot represents the percentage of cells expressing the target gene within a specific cluster (Percent Expressed), while the color gradient indicates the scaled average expression level of the gene (Average Expression).

Supplementary Figure 3. Spatial transcriptomics quality control and structural mapping.

(A) Spatial feature plots mapping key quality control metrics—including Genes per Spot, UMIs per Spot, Mitochondrial percentage (%), and Ribosomal percentage (%)—across the spatial capture areas for individual Control (C1, C2) and HT (HT1, HT2, HT3) tissue sections.

(B) Spatial mapping depicting the post-QC tissue structure and spot distribution overlaid onto the corresponding histological sections for each sample.

Supplementary Figure 4. Transcriptomic mapping reveals the upregulation of the necroptosis pathway in Hashimoto’s thyroiditis.

KEGG pathway maps for Necroptosis (hsa04217) rendered by Pathview. Differentially expressed genes (DEGs) identified from two independent datasets, GSE138198 (A) and HRA001684 (B), are mapped onto the pathway. Red nodes indicate upregulated genes, and green nodes indicate downregulated genes in HT compared to Controls. The scale bar represents the log2 fold change.

Supplementary Figure 5. Spatial mapping of module scores in HT sections.

Spatial distributions of the TFC differentiation score, inflammatory cytokine score, RIPK3-MLKL execution score, and RIPK1 related score are shown in P3 from GSE230424 (A) and in HT1-HT3 from GSE248205 (B-D).

Supplementary Figure 6. Spot-level correlation analysis of spatial module scores in HT sections.

Spearman correlation analyses were performed within each HT section from GSE248205 (HT1-HT3; A-C) and GSE230424 (P3-P4; D-E). Each panel shows correlations between the RIPK3-MLKL execution score and inflammatory cytokine, TFC differentiation, and RIPK1 related scores, as well as the correlation between the RIPK1 related score and inflammatory cytokine score.

Supplementary Figure 7. Exploratory performance of necroptosis- and inflammation-related markers in distinguishing HT from controls.

(A) Receiver Operating Characteristic (ROC) curves evaluating the diagnostic accuracy of key genes (IFNG, TNFA, JAK2, STAT1, IRF1, RIPK3, ZBP1, MLKL) in distinguishing HT patients from controls. The analysis includes data from the local cohort validation (rt-PCR, pink), and two external transcriptomic datasets: HRA001684 (green) and GSE29315 (purple). The Area Under the Curve (AUC) values are indicated for each dataset. (B) ROC curves for serum inflammatory cytokines (IL-6, IL-1α, IL-1β) based on ELISA data from the local cohort.

Supplementary Figure 8. Associations among necroptosis-related molecules, inflammatory cytokines, thyroid differentiation markers, and clinical indicators.

(A) Heatmap illustrating the Pearson correlation coefficients between necroptosis-related genes and inflammatory mediators in HT thyroid tissues. (B) Correlation matrix of serum cytokine levels (IL-33, IL-1α, IL-1β, IL-6). (C) Comprehensive heatmap displaying the correlation between the "Necroptosis/Inflammation Module" (vertical axis: ZBP1, IL33, IFNG, JAK2, STAT1, IL1B, IRF1, IL6, TNF, RIPK3, MLKL, TRAF5) and "Thyroid Function/Differentiation Module" (horizontal axis: TG, TPO, TSHR, NKX2-1, SLC26A4, DIO1/2, etc.). Statistical significance is indicated as *p < 0.05, **p < 0.01, ***p < 0.001, ****p < 0.0001, ns: not significant.
